# Insomnia

**Published:** 1889-03-02

**Authors:** William Moore

**Affiliations:** late Surgeon-General with the Government of Bombay


### Insomnia.

By Sir William Moore, K.C.I.E., late Surgeon-General with the Government of Bombay.

Insomnia, or sleeplessness, is not only a very annoying but oftentimes a very serious ailment—annoying in its present aspects, serious in the near future. One or two nights of sleeplessness would not, of course, much disturb the average robust individual, but when *pervigilium* is long continued the results are debility, exhaustion, nervousness, mental disquietude, palpitation of the heart, and hypochondriasis. Hood truly observed that "they must be wretched who cannot sleep when God Himself draws the curtain;" and, indeed, insomnia is often a symptom of, and frequently a prelude to, insanity, especially *dementia*. There may be no desire to sleep, or there may be every wish to sleep and inability to do so. In other instances there is a dread of going to sleep. Or a person may be drowsy in the daytime, but unable to sleep at night. Or after a sleepless night, he may be sleepy when he ought to get up. These two last phases are perhaps the most distressing forms of the malady, which is thus brought home, not only during the night, but in the day also. It is a traveller's tale, that when a desert Arab cannot sleep his sheikh makes use of him by setting him to watch the camels. In civilised life we cannot thus utilise the victim of insomnia, so are obliged to seek a remedy. Unfortunately we have not arrived at "a thousand years hence," of which future period Nunsowe Green, Esquire, describes the sleeping arrangements. The person wishing to sleep is to lie down in a head-to-foot magnetic current, which composes at once to sleep, while a clock regulation by arrest and reverse of the composing current after so many hours causes immediate awakening. Our present-century plan is a much more "roundabout" procedure, and involves as a first principle finding out the cause of the sleeplessness; for it is necessary that every case should be treated on its own merits.

Sleeplessness is most common to those of nervous temperaments, and especially if their pursuits involve brain work. The natural, hereditary temperament cannot be much altered, except by the callousness of age. Many things act on a person of nervous temperament to a much greater degree and with more force than on others of a more phlegmatic nature. Therefore, to attain that sleep which "knits the ravell'd sleeve of care," it is necessary for those of nervous temperament to avoid all excitements which, from experience, they know are followed by sleeplessness, and especially brain work at night. The work of the day should be dismissed from the mind. Care, anxiety, worry must be banished. Men whose minds are ill at ease from anxiety, sorrow, remorse, have their sleep murdered like Macbeth. All this, however, is not easy of accomplishment. Everyone cannot arrive at that state of stern and passive repose of mind which, according to Epictetus, constitutes happiness. Yet something of this is essentially required to combat nervous sleeplessness. If this cannot be attained, recourse must be had to the palliatives mentioned below, when the nervous individual courts Nature's sweet restorer. A fertile cause of sleeplessness is *anœmia*. Anæmia itself, especially in those of nervous temperament, produces an irritable condition. The anæmic person becomes capricious and irritable—impressions too feeble to annoy the healthy person harrassing the anæmic. But this anæmia, which has sleeplessness as one of its symptoms, may be excited by a variety of causes, among which are insufficient diet, food deficient in certain qualities, dark rooms, want of exercise, prolonged fatigue, mental or physical, constipation, too much tobacco-smoking, bad hygiene, an unsuspected scorbutic or specific taint, tapeworm, and in women over-nursing. When tone is thus lowered a moderate supper of well-cooked and nutritious food is often beneficial, but at the same time treatment is required for the cause of the anmæia. Perhaps the most common cause of insomnia is dyspepsia in one or other of its protean forms, which must be treated both medically and hygienically. Here we need only say that the sleepless dyspeptic should not go to bed with an undigested meal in the stomach, and should avoid coffee, tea, and usually tobacco at night. In the absence of any special cause of insomnia as above, it may arise from close, unventilated rooms, the remedy for which is obvious. Or it may arise from cold feet, the remedy for which is also obvious. Dr. Stewart, in his work on temperaments, observes: "Probably Mr. Gladstone takes to felling trees to ward off the sleeplessness for which his physician had to send him to the sunny South a few years ago." Suppressed or latent gout has been credited as a cause, and this should be held in mind. Sleeplessness from alcohol culminates in delirium tremens, but usually long before delirium occurs Morpheus deserts the couch of the votary of alcohol. Affections of the heart will induce sleeplessness, and sleeplessness will induce affections of the heart. When the exhaustion from prolonged sleeplessness obtains it is sometimes demonstrated through the heart in the shape of palpitation and intermittent action, bringing many evils in their train. An uncomfortable bed often prevents repose. According to Pliny, the Romans, wise in their generation, had comfortable feather couches. But straw was used, even in the royal chambers of England, so late as the close of the thirteenth century. Now, in addition to feathers and straw, we have beds of horsehair, iron, and of other material, so that the sufferers from sleeplessness has a large choice. For a long time beds were laid on the ground, until elevation was introduced from the East. A difference of either material or of elevation is often sufficient to cause sleeplessness. But a strange bed is worse. It rarely fits right, it often smells strange, one settles down at the wrong angle; if a spring bed it cracks at odd times, if a feather bed there are lumps where no lumps should be. One takes to wondering who slept on it last, and whether the last occupant died of a fever, and then—goodbye to sleep!

A strange room is almost as bad. The corner which should be light is dark, and the corner which should be dark is light. Strange shadows come up to look at us, and strange noises are heard. If the occupant believes in spiritual manifestations, goodbye to sleep. Monotonous murmurs have long been noted as soporific. The Æolian harp placed in an open window might be beneficial in tropical climates, or even on the Riviera, but is scarcely suitable to our inclement seasons. Virgil expatiates on the murmur of bees, but bees in our bed-rooms would not be altogether pleasant companions, even if they murmured by night. Neither can we well introduce a murmuring brook, or even a stream of running water through our bed-rooms. Shakespeare says, "the crickets sing, and man's o'er laboured sense repairs itself to rest;" but, in our experience, the British cricket makes a chirrup anything but conducive to repose. Had Byron been nervous or anæmic, he would never have written—except by poet's license—"'tis sweet to hear the watch-dog's honest bark." And had Sir Edwin Arnold been nervous or anæmic, he would not have written about the musical sound of the Indian village well, which of all sleep-dispelling agencies is one of the most discordant. When no particular cause of insomnia can be discovered, there are many palliatives which may be tried. "After supper walk a mile" is not bad advice, although no supper, except perhaps for the anæmic, is better. Asclepiades used to be rocked to sleep, but to this we suppose our children of large growth of the present day would not submit. Putting the feet in warm water before retiring may do good. Regular hours, so that the force of habit may be enlisted in favour of sleep, is almost a necessity. A glass of cold water taken immediately before going to bed will sometimes induce repose, but a "night cap" in the form of a stimulant is only of temporary benefit, and not advisable. Opiates, as chloral or morphia, or even bromide of potassium, should never be used, for a habit is thereby formed which may be difficult to overcome, and the sleep caused by such agents is not that natural repose which is the "chief nourisher in life's feast." Moreover such agents require to be increased in dose, and this becomes injurious to the system. As before mentioned, a thousand years hence, we are to be put to sleep by an electric current. In the meantime, there may be something in the advice given, to sleep with the head to the north, for there are electric currents constantly passing, which it may not be well to oppose with the breath of our bodies. It is also better to have the bed towards the centre of the room, or at least not close to, or broadside on to, a wall. Constantly repeating numerals, or X, Y, Z, are popular soporifics, but we do not recollect hearing of much benefit resulting therefrom. Thinking of some pleasant occurrence of "auld lang syne" is a better soporific. Getting up and taking a walk up and down the room is sometimes successful, why we do not know, although a physiological explanation has been attempted. In conclusion, we may remark that "sleep not on your back in the posture of a dead man," was the advice given by both Confucius and Hippocrates.

